# Chitosan Hydrogels Crosslinked with Oxidized Sucrose for Antimicrobial Applications

**DOI:** 10.3390/gels9100786

**Published:** 2023-09-29

**Authors:** Sayaka Fujita, Hijiri Takeda, Junki Noda, Haruki Wakamori, Hiroyuki Kono

**Affiliations:** 1 Division of Applied Chemistry and Biochemistry, National Institute of Technology, Tomakomai College, Nishikioka 443, Tomakomai 059-1275, Hokkaido, Japan; fujita@tomakomai-ct.ac.jp (S.F.);; 2 Hokkaido Soda Co., Ltd., Numanohata 134-122, Tomakomai 059-1364, Hokkaido, Japan

**Keywords:** chitosan, oxidized sucrose, polysaccharide hydrogel, antimicrobial activities

## Abstract

Oxidized sucrose (OS) reacts with amino-group-containing polysaccharides, including chitosan, without catalyst, resulting in hydrogels entirely composed of carbohydrates. The presence of imine bonds with low structural stabilities and unreacted aldehydes in the structures of these hydrogels hinder their application as biomaterials. Therefore, herein, the chitosan hydrogels (CTSGs) obtained after the crosslinking of chitosan with OS were reduced using sodium borohydride to convert imine bonds to secondary amines and aldehydes to alcohols. The structures of CTSGs were comprehensively characterized using Fourier transform infrared and ^13^C nuclear magnetic resonance spectroscopies, and the results implied that the degree of crosslinking (CR) depended on the OS feed amount used during CTSG preparation. The properties of CTSGs were significantly dependent on CR; with an increase in CR, the thermal stabilities and dynamic moduli of CTSGs increased, whereas their swelling properties decreased. CTSGs exhibited antimicrobial properties against the gram-negative bacterium *Escherichia coli*, and their performances were also dependent on CR. The results indicated the potentials of CTSGs completely based on carbohydrates as antimicrobial hydrogels for various medical and pharmaceutical applications. We believe that this study will contribute to the development of hydrogels for application in the food, medical, and pharmaceutical fields.

## 1. Introduction

In recent years, chitosan has attracted attention as a very useful green biopolymer. Chitosan is derived from chitin obtained from the shells of crabs, shrimps, and insects, and it is a renewable polymer that is abundant in nature and the second highest produced polysaccharide after cellulose. Chitosan is achieved via the alkaline deacetylation of chitin consisting of β-(1,4)-*N*-acetylglucosamine (GlcNAc) residues; its structure is composed of β-(1,4)-glucosamine (GlcN) residues and little or no GlcNAc residues. Chitosan exhibits excellent biocompatibility, biodegradability, low immunogenicity, ecological safety, and non-toxicity [1,2]. The numerous amino groups on the chitosan backbone can be protonated at pH < 6.5 (the pKa value of the amino group in chitosan is approximately 6.5), rendering chitosan to behave as a polycation. As a polycation, chitosan demonstrates antimicrobial properties due to the electrostatic interaction between the positive surface charges of the amino groups in GlcN and negatively charged microbial cell membranes [2]. These properties have facilitated the application of chitosan in food-related, medical, and pharmaceutical processes such as food preservation [3], disease control in agricultural crops [4], wound dressing [5], and skin care [6]. However, the practical applications of chitosan are limited by its inferior water solubility caused by inter- and intra-molecular hydrogen bonds and low mechanical strength arising from low crystallinity in comparison to those of cellulose and chitin. For example, chitosan-based tissue engineering scaffolds can easily break and fail to support tissue engraftment. Additionally, chitosan films and membranes are brittle and not ductile enough, thereby fracturing during wound healing.

Hydrogels [7], microparticles [8], three-dimensional (3D) porous sponges [9], and blends of chitosan with other polymers [10] have been investigated to broaden the applications of chitosan. Hydrogels are 3D network structures of polymers with crosslinked molecular chains, which can absorb and retain water. The retained water does not leak out under pressure. Hydrogels contain extremely large amounts of water (>90%) and are also used as medical materials because their water content composition is similar to those of human tissues. For instance, they are employed as hemostatic and anti-adhesive agents for affected areas [11], drug delivery system (DDS) carriers [12], and tissue scaffolds for regenerative medicine [13]. In the preparation of chitosan-based hydrogels, glutaraldehyde [14], epichlorohydrin [15], and ethylene glycol diglycidyl ether [16] are commonly used as crosslinking agents. These materials are highly toxic, and the resulting hydrogels are unsuitable for biological applications. In contrast, genipin is a naturally occurring crosslinking agent; nevertheless, its high cost renders its sustainable use difficult for economic reasons [17]. Oxidized sucrose (OS) is a non-toxic and inexpensive crosslinking agent. It is obtained through the oxidation of sucrose with periodate and has a polar backbone structure comprising hydroxyl groups and glycosidic bonds; moreover, unlike glutaraldehyde and formaldehyde, OS is nonvolatile and not environmentally toxic [18]. Furthermore, in vitro cell culture studies of protein scaffolds prepared using OS have demonstrated the biocompatibility of OS, rendering it an environmentally friendly and safe crosslinking agent [19]. During the oxidation of sucrose, the C3–C4 bond of the fructose residue and C2–C3 or C3–C4 bond of the glucose residue are oxidatively cleaved. Thus, OS consists of four aldehydes. Via these four aldehyde moieties, it can crosslink polysaccharides such as starch [20] and proteins including collagen [21] and keratin [22].

Previously, we have fabricated a hydrogel of carboxymethyl chitosan (CMC) using OS as a crosslinking agent [23]. The reaction between the amino group of CMC and aldehyde moieties of OS occurred in an aqueous solution at room temperature without catalyst, and corresponding hydrogels were readily obtained. The obtained CMC hydrogels swelled in natural region and their absorbency is attributed to the osmotic pressure generated by the free sodium cations and the electrostatic repulsion between CMC molecular chains due to the presence of carboxylate anions. CMC hydrogels do not sufficiently swell in acidic regions where the protonation of amino groups occurs, and are not expected to exhibit antimicrobial activities. In this study, chitosan was crosslinked with OS to synthesize chitosan hydrogels (CTSGs). The use of chitosan instead of CMC could provide the hydrogels that swell in acidic regions and possess antimicrobial activities. Crosslinking between the amino groups of chitosan and OS formed imine bonds. Moreover, the aldehydes in OS that were not used in crosslinking were retained in the structures of CTSGs. The imine bond is structurally unstable and unreacted aldehyde may further react with the amino group of chitosan. The properties of hydrogels strongly depend on the crosslinking density of the resultant polymer; e.g., an increase in the crosslinking density generally leads to a decrease in water absorbency. Moreover, the antimicrobial activities depend on the number of amino groups. This means that a decrease in the number of amino groups leads to a decrease in antimicrobial activities. For their application as biomaterials, it is necessary to prevent the structure and property changes in CTSGs due to the cleavage of imine bonds and the reaction between unreacted aldehyde and the amino groups. Therefore, CTSGs were reduced using sodium borohydride (NaBH_4_) to convert the imine bonds to secondary amines and aldehydes to alcohols, providing structurally stable hydrogels. The structures, rheological and thermal properties, swelling behaviors, and antimicrobial activities of the resulting CTSGs were investigated. Furthermore, based on the data obtained via these analyses, the structure–function relationships of CTSGs were examined with the aim of contributing to the development of hydrogels for application in the food, medical, and pharmaceutical fields.

## 2. Results and Discussion

### 2.1. Preparation of CTSGs

**Figure 1:**
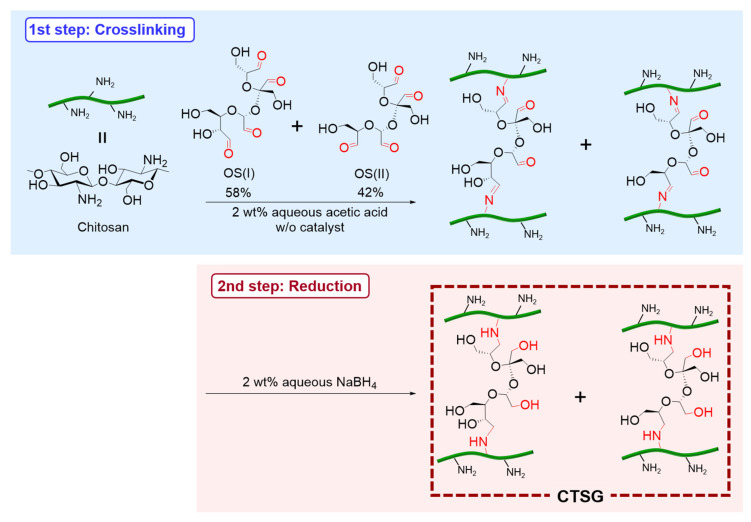
Preparation of chitosan hydrogels (CTSGs) using oxidized sucrose (OS) as a crosslinking agent. CTSGs were synthesized via two steps: crosslinking of chitosan with OS (top) and reduction of aldehydes and imines by NaBH_4_ (bottom).

Before the synthesis of CTSGs, OS was fabricated according to a previous study [23]. During the oxidation of sucrose with sodium periodate (NaIO_4_), the C3–C4 bond of the fructose residue and C2–C3 or C3–C4 bond of the glucose residue were oxidatively cleaved, yielding a mixture containing two structures of OS (namely, OS(I) and OS(II)) (Figure 1). The composition of OS was determined to be 52 mol% OS(I) and 48 mol% OS(II) via quantitative ^13^C nuclear magnetic resonance (NMR) spectroscopy. Considering that the prepared OS is a mixture of OS(I) and OS(II), the average molecular weight and aldehyde content of OS were evaluated (325 g mol^–1^ and 12.3 mmol g^–1^), respectively.

Subsequently, OS was used as a crosslinking agent to prepare CTSGs. CTSGs were fabricated via two steps: crosslinking and reduction (Figure 1). The crosslinking of chitosan was conducted using OS in aqueous acetic acid without a catalyst at 298 K for 24 h (Figure 1 top). A series of three hydrogels (CTSG 1–3) (Table 1) were prepared by varying the initial OS:chitosan feed ratio. The four aldehyde groups of OS can react with the amine groups of chitosan to form imine bonds. Therefore, the molar aldehyde contents of OS were set to 0.2, 0.3, and 0.5 times those of the free amino groups of chitosan. Although the chitosan solution demonstrated no change immediately after the addition of OS, it became extensively viscous over time and the morphology of the mixture gradually transformed from a solution to a gel. After 24 h of reaction, a clear yellow gel was acquired. The gel turned more yellowish with an increase in the feed amount of OS. CTSGs containing imine bonds formed using crosslinking agents, such as glutaraldehyde, are often yellowish and become more yellowish with an increase in the number of imine bonds [24,25]. Therefore, the change in the color of the gel with an increase in the feed amount of OS suggested an increase in the amount of OS in the gel. The yellow gel was crushed and employed for the second step, that is, reduction. The imine bonds produced by the crosslinking of chitosan with OS demonstrate low structural stabilities. The gel also comprises unreacted aldehydes that may further react with the amino groups of chitosan, thereby altering the structure and properties of CTSG. Additionally, if CTSG is used in vivo, unreacted aldehydes in CTSG can react with the amino groups of proteins, which hinders the application of CTSG as biomaterials. Thus, during reduction, the imine bonds and unreacted aldehyde moieties in the yellow gel were converted to secondary amines and alcohols, respectively. The crushed yellow gels were immersed in 2 wt% aqueous NaBH_4_ for 24 h. The gels changed from yellow to colorless during reduction. The obtained CTSGs were purified by washing with water and subjected to dialysis to remove unreacted OS. Finally, lyophilization was performed to obtain CTSG 1–3 as white cotton-like hygroscopic solids.

### 2.2. Structural Characterizations of CTSGs

**Figure 2:**
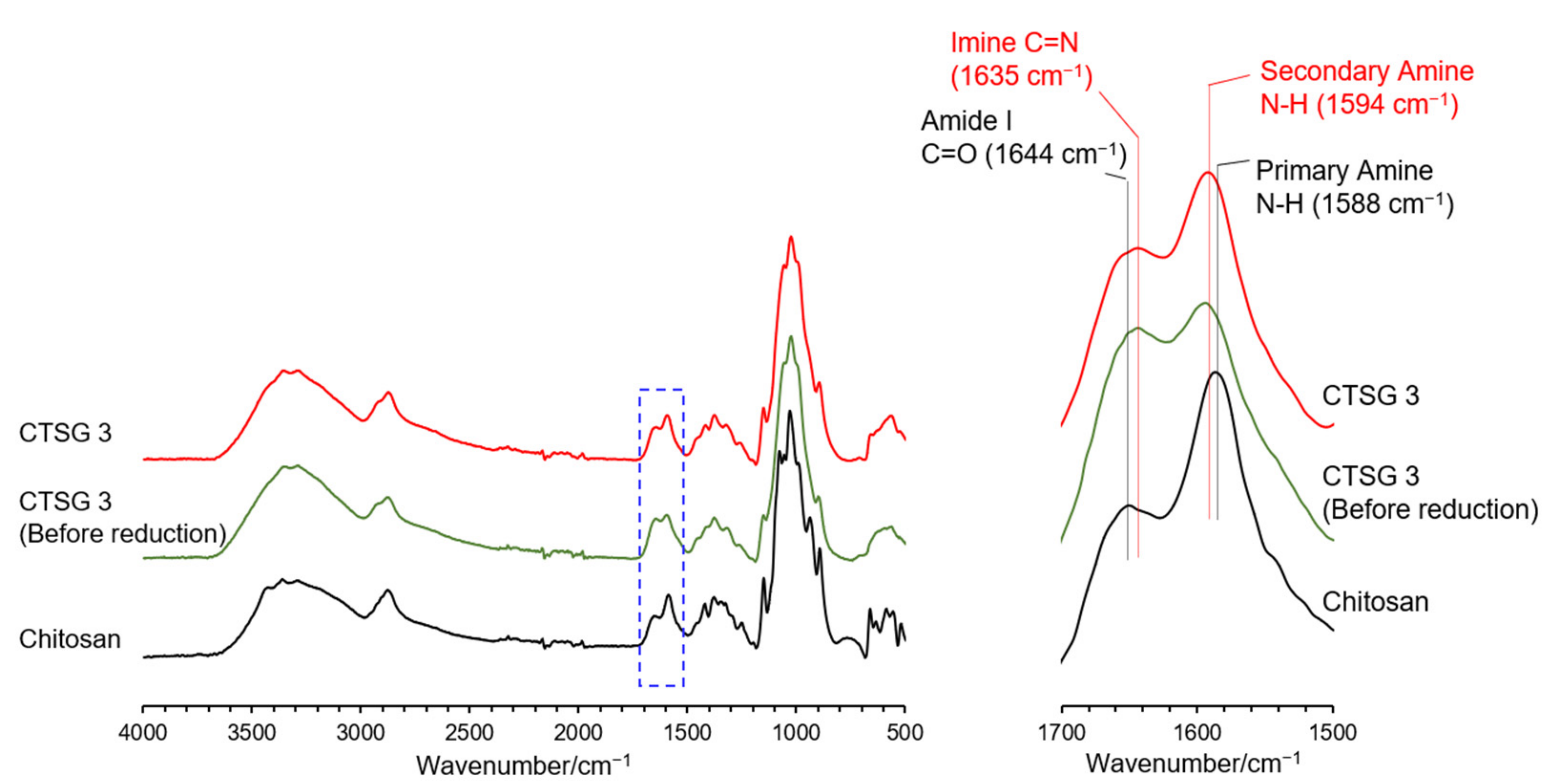
Full (**left**) and expanded (**right**) FTIR spectra of CTSG 3 before and after reduction and chitosan.

Figure 2 shows the Fourier transform infrared (FTIR) spectra of CTSG 3 before and after reduction and the starting material chitosan. In the spectrum of chitosan, the broad absorption band at 3317 cm^−1^ corresponded to N–H and O–H stretching vibrations, and the absorption band at 2892 cm^−1^ was assigned to the C–H stretching vibration [26]. The characteristic absorption bands corresponding to the C=O stretching vibration (amide I) and N–H bending vibration (primary amine) were observed at 1644 and 1588 cm^−1^, respectively [26]. Compared with the FTIR spectrum of chitosan, the FTIR spectrum of CTSG before reduction exhibited a new absorption band related to the C=N stretching vibration of the imine group (1635 cm^−1^) [23,27] as a shoulder of the amide I absorption band. This implied that the aldehyde groups of OS reacted with the amine groups of chitosan to form imine bonds. As the imine bonds were converted to amine moieties via reduction, the C=N absorption band should be absent in the spectrum of CTSG 3 after reduction. Nevertheless, this could not be clearly noticed due to the overlap of the imine absorption band with the amide I absorption band. The FTIR spectrum of CTSG 3 after reduction demonstrated the absorption band of secondary amine at 1594 cm^−1^ [28], suggesting the reduction of the imine bond to secondary amine. The absorption band of the aldehyde group (C=O stretching) was not observed at 1710 cm^−^^1^ [23,29] in the spectra of CTSG before and after reduction. The FTIR spectra did not clearly confirm the presence of aldehydes in CTSG and the reduction of aldehydes.

**Figure 3:**
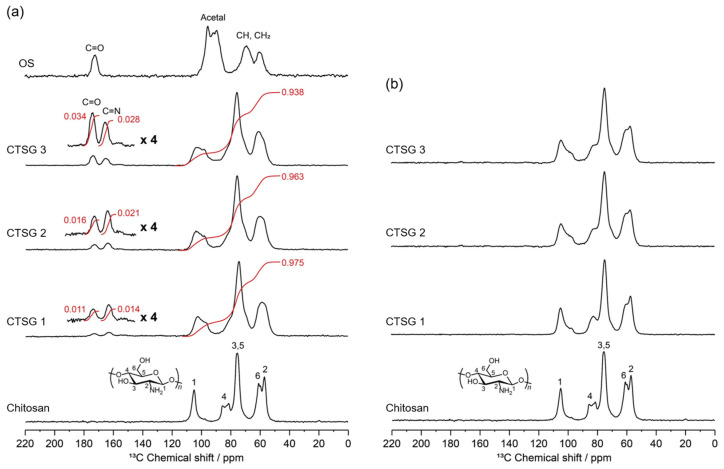
Quantitative solid-state ^13^C NMR spectra of CTSG 1–3 (**a**) before and (**b**) after reduction. The spectra of chitosan and OS are also shown.

Figure 3 depicts the quantitative solid-state ^13^C NMR spectra of CTSG 1–3 before and after reduction. In the NMR spectrum of chitosan, the broad resonances at 105, 87, 78, 63, and 56 ppm were attributed to the pyranose carbon C1, C4, overlap of C3 and C5, C6, and C2 in the chitosan backbone, respectively [30]. In the spectrum of OS, the resonances of the carbon moieties of acetal, namely, CH and CH_2_, and the carbonyl carbons of the aldehyde groups were detected at 100–50 and 173 ppm, respectively. Compared with the spectrum of chitosan, the spectra of CTSGs exhibited new resonances at 168 and 173 ppm, which were assigned to imine carbons and carbonyl carbons of the aldehyde groups, respectively [23]. The existence of unreacted aldehydes and imines in the structures of CTSGs could not be distinctly verified via the FTIR spectra, whereas it was corroborated by the ^13^C NMR spectra. These results indicated that OS reacted with the amino groups of chitosan. In the spectrum of CTSG after reduction, the resonances of the carbonyl carbons of the imine and aldehyde moieties were completely absent (Figure 3b), implying that the imine and aldehyde moieties were reduced to amine and alcohol moieties, respectively.

To estimate the amount of OS crosslinked to chitosan in CTSGs, the integral values of imine carbon (*I_C=N_*), carbonyl carbon of aldehyde (*I_C=O_*), and other carbon resonances (*I_others_*) were determined (Figure 3a), and the sum of the integral values of all resonances was 1 (*I_C=N_* + *I_C=O_* + *I_others_* = 1). The degrees of crosslinking (CRs) of CTSGs were calculated by speculating that OS introduced into chitosan was not dissociated by reduction. At first, the number of OS molecules reacted per glucosamine residue of chitosan (*n_OS_*) was determined. The four aldehyde groups of OS reacted with chitosan to generate imine bonds, and CTSGs demonstrated four structures, as described hereinafter. The crosslinking between chitosan and OS resulted in CTSGs with two imine bonds and two unreacted aldehydes (Figure 1). The graft reaction led to the formation of one imine bond and three unreacted aldehydes. Moreover, a tri-bond with three imine bonds and one unreacted aldehyde and a tetra-bond with only four imine bonds can also be produced; however, the probability of the generation of these bonds is substantially less than that of the formation of crosslinking and grafting bonds. In all these structures, the sum of the carbon atoms of aldehydes and imine carbon atoms in the OS linkage is always four and remains unchanged. Thus, the number of carbon atoms of aldehyde and imine in CTSG was 4*n_OS_*. Considering that the composition of OS is OS(I) and (II), OS used in crosslinking possesses an average of 11.6 carbon atoms per molecule, among which four carbon atoms are carbonyl carbons of aldehydes. Excluding the number of carbons of the aldehyde and imine groups, the number of carbons per OS-introduced glucosamine residue in CTSG was 6 + 7.6*n_OS_*. The relationship between *n_OS_* and the integral values of resonances was established using Equation (1):

(1) $$\frac{4n_{OS}}{6+7.6n_{OS}}=\frac{I_{C=O}+{\;I}_{C=N}}{I_{others}}$$

The CR of a CTSG is defined as the number of imine bonds (which are converted to amine bonds after reduction) formed between chitosan and OS per glucosamine residue and determined using Equation (2):

(2) $$CR=4n_{OS}\frac{I_{C=N}}{I_{C=O}+I_{C=N}}$$

The determined CR and *n_OS_* for each CTSG are provided in Table 1. CRs and *n_OS_* values for CTSG 1, 2, and 3 were 0.09, 0.14, and 0.20 and 0.04, 0.06, and 0.11, respectively. Clearly, CR and *n_OS_* increased with an increase in the OS:chitosan feed ratio during CTSG synthesis. When one molecule of OS as a crosslinked structure was introduced into chitosan, the number of formed imine bonds was two. That is, a CR:*n_OS_* ratio of two indicates that the introduced OS is a crosslinked structure. CR:*n_OS_* ratios for CTSG 1, 2, and 3 were 2.2, 2.3, and 1.8, respectively, which were close to 2. Thus, most of the reacted OS molecules were incorporated into the chitosan molecule as crosslinked structures.

### 2.3. Thermal Properties of CTSG

**Figure 4:**
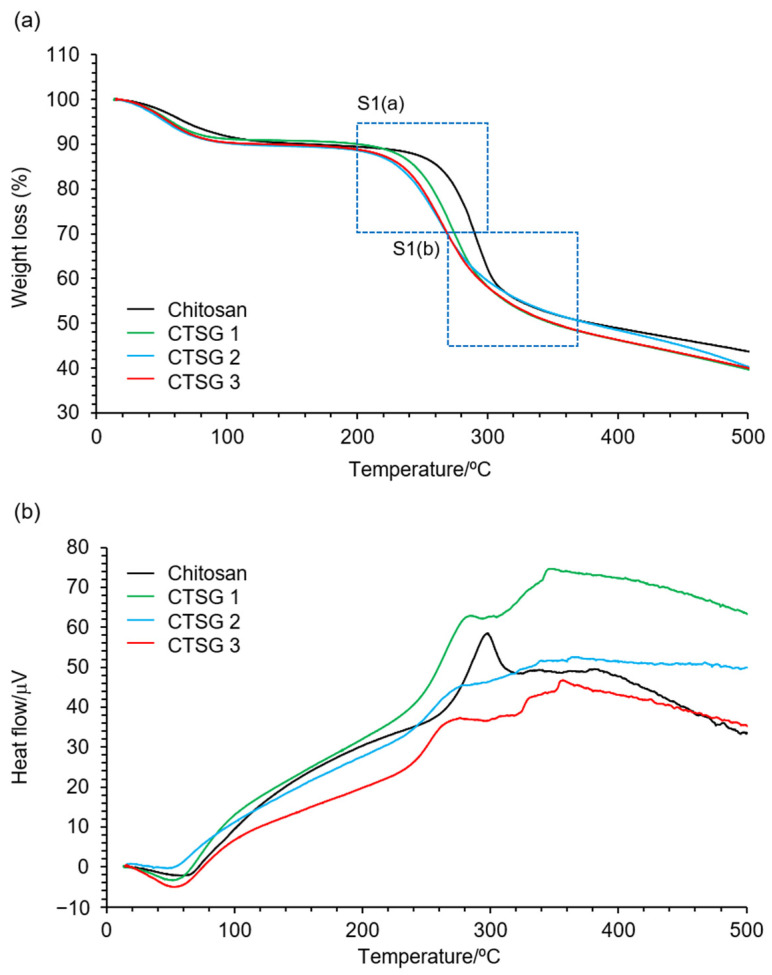
(**a**) Thermogravimetric (TG) and (**b**) differential thermogravimetric analysis (DTA) curves for CTSG 1–3 and chitosan. Magnified TG curves enclosed by dashed lines are shown in Figure S1a,b.

Figure 4a,b depicts the thermogravimetric (TG)/differential thermogravimetric analysis (DTA) curves of CTSG 1–3 and chitosan. The TG curve of chitosan demonstrated three weight-loss stages. During the first stage, a weight loss of approximately 11.5% occurred until 190 °C, attributed to the vaporization of physically absorbed and intermolecular hydrogen-bonded water [31]. The second stage of weight loss took place in the range of 205–310 °C and was ascribed to the depolymerization/decomposition of polymer chains and cleavage of glycosidic linkages [32]. The last stage of weight loss occurred at temperatures higher than 310 °C, corresponding to thermal destruction of the pyranose ring and decomposition of residual carbon [33]. These thermal behaviors were confirmed by the presence of an endothermic peak at 67 °C and exothermic peaks at 298 and 380 °C in the DTA curve. The TG curves of CTSG also demonstrated three weight-loss stages similar to those of chitosan.

Figure S1a,b shows the initial and final stages of the second stage of thermal decomposition. The initial (*T_i_*) and final (*T_f_*) decomposition temperatures for the second stage of degradation of chitosan and CTSG 1–3 are also depicted. The *T_i_* and *T_f_* of chitosan were determined to be 264 and 308 °C, respectively. In the cases of CTSG 1–3, the *T_i_* values decreased and *T_f_* values increased with an increase in CR. Generally, the incorporation of functional groups into polysaccharides causes the loss of interactions (for example, hydrogen bonding) between molecular chains, which decreases the thermal stabilities of polysaccharides [34,35]. The decrease in the *T_i_* values of CTSG 1–3 was attributed to the decrease in the number of hydrogen bonds between molecular chains owing to the introduction of OS into the molecular chains of chitosan. The decrease in *T_i_* with an increase in CR was explained by the loss of thermal stability of chitosan originating from hydrogen bonds upon the crosslinking of chitosan with OS. In contrast, the chemically crosslinked structures strengthened the molecular structures of CTSG 1–3 and increased the corresponding *T_f_* values. This tendency was reported for a carboxymethyl chitosan hydrogel fabricated using OS as a crosslinking agent. In conclusion, the crosslinking of chitosan with OS decreased the interaction between the molecular chains of chitosan and resulted in CTSG 1–3 with high structural stabilities.

### 2.4. Water Absorbencies of CTSG 1–3 with Respect to pH

**Figure 5:**
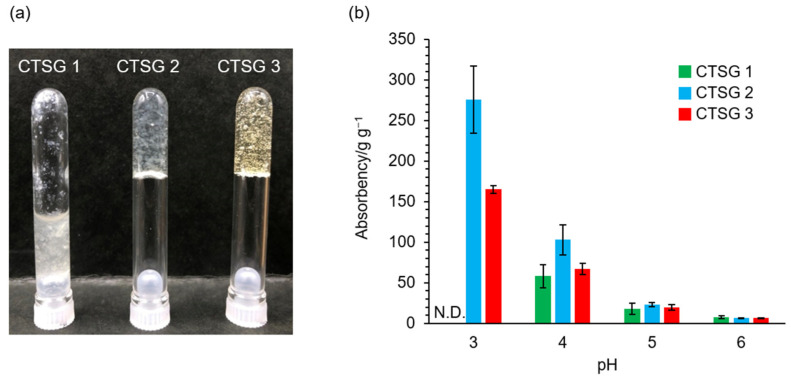
(**a**) Image showing swollen CTSG 1–3 in 20 mM acetate buffer (pH = 3). (**b**) Effects of pH on the absorbencies of CTSGs.

Images of swollen CTSG 1–3 in 20 mM acetate buffer (pH = 3) are shown in Figure 5a. When placed in acetic acid buffer, lyophilized CTSG 2 and 3 immediately absorbed water and turned transparent, whereas CTSG 1 remained in a fluid state. The water absorbencies of CTSG 1–3 at pH = 3–6 are depicted in Figure 5b. At pH = 3, CTSG 1 was very fluid, CTSG 1 and unabsorbed excess solution could not be separated by centrifugation, and its water absorbency could not be determined. Except for the case of CTSG 1, the water absorbencies of all CTSGs decreased with an increase in pH. The water absorbencies of chitosan-based hydrogels are caused by the protonation of the amino groups on the backbone of chitosan [36]. Under acidic conditions, as the dominant charged species in these hydrogels are protonated amino groups, the intermolecular network structure expands due to the electrostatic repulsion between protonated amino groups, resulting in high water absorption. Under the near-natural conditions of pH = 5 and 6, the amino groups are deprotonated, the expansion of the network is suppressed, and water absorption is reduced. The water absorbencies of CTSG 2 and 3 decreased with an increase in CR. The highly crosslinked structures suppressed the expansions of chitosan molecules in CTSG 2 and 3, leading to lower water absorption.

### 2.5. Rheological Properties of CTSGs

**Figure 6:**
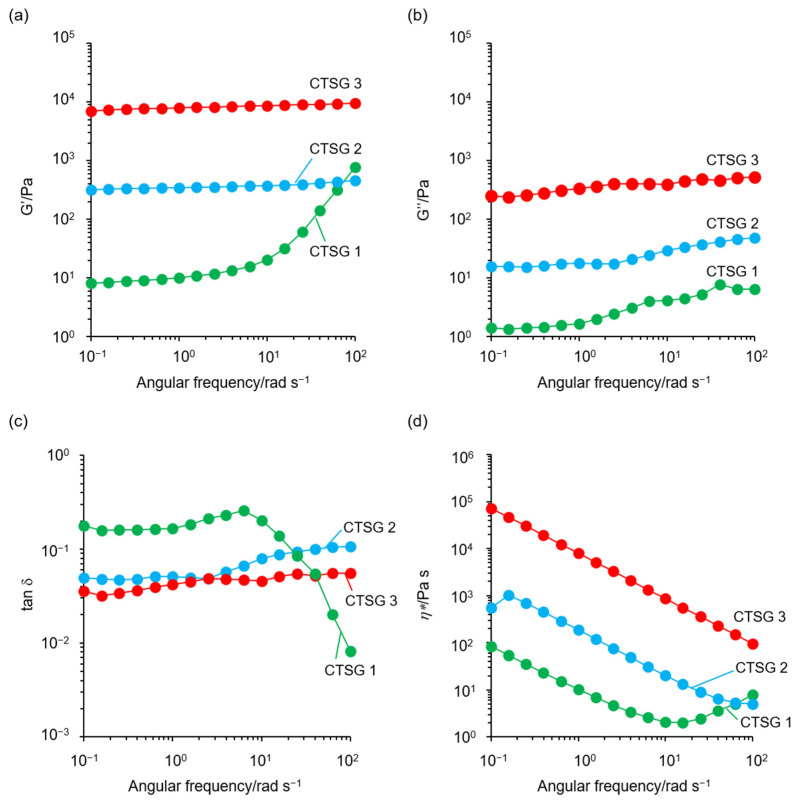
Rheological properties of CTSG 1–3 swollen and saturated with 20 mM acetate buffer (pH = 4) at 298 K: (**a**) storage modulus (G′), (**b**) loss modulus (G″), (**c**) tan δ (= G″/G′), and (**d**) complex viscosity (η*).

Figure 6 shows the plots of the storage (G′) and loss (G″) moduli, tan δ, and complex viscosities (ƞ*) against the angular frequency sweeps for CTSGs swollen and saturated with acetate buffer with pH = 4. The G′ values of all CTSGs were always higher than their G″ values, and no crossover points (tan δ = 1) were noticed. The tan δ values of CTSGs were in the range of 0.008–0.25, indicating that the elastic properties of CTSGs were superior to the viscous properties in the dynamic viscoelastic behaviors of CTSGs. For CTSG 2 and 3, the G′ and G″ values were almost independent of the frequency characteristic of the permanent gel network. In contrast, the G′ and G″ values of CTSG 1 exhibited drastic changes in the frequency dependence over 10 rad s^–1^, implying that CTSG 1 exhibited the viscoelastic behavior of a weak gel [37]. Additionally, G′, G″, and η* increased with an increase in CR. These observations indicated that the viscoelastic behaviors of CTSGs were considerably dependent on CR and the addition of a large amount of OS to the reaction mixture to produce CTSGs significantly increased G′, G″, and η*.

### 2.6. Antimicrobial Activities of CTSGs

**Figure 7:**
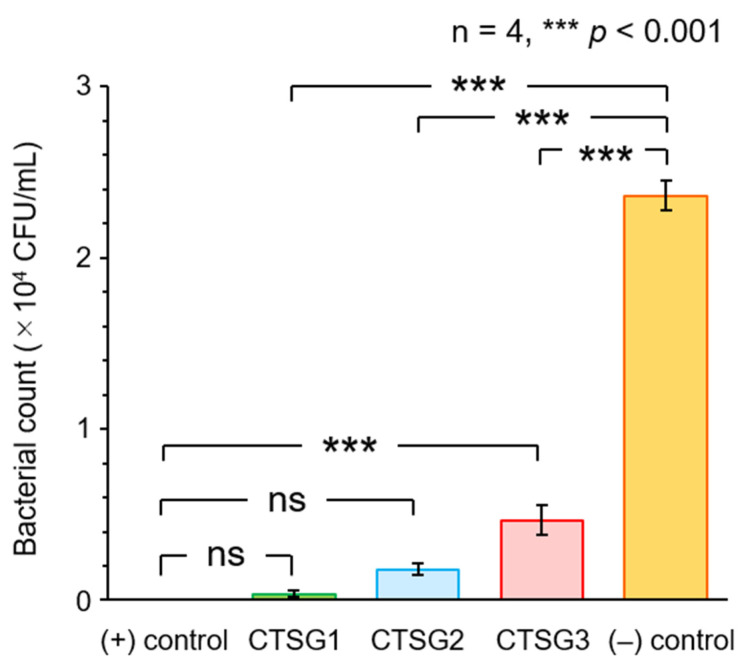
Antimicrobial activities of CTSG 1–3 against *Escherichia coli*, with chitosan and no sample as positive and negative controls, respectively. The columns and attached bars represent the means of the results of four identical samples and their standard errors. Asterisks and ns correspond to significant differences and non-significant differences between the results of the samples determined using statistical analysis (Tukey’s test).

The antimicrobial activities of CTSGs against *Escherichia coli* (*E. coli*) were assessed by quantifying the number of colonies (colony-forming unit (CFU)/mL) of *E. coli* after the incubation of *E. coli* with CTSGs in Lysogeny Broth (LB) medium for 3 h (Figure 7). The cultured LB medium was incubated on standard method agar plates at 310 K for 24 h, and then, the number of colonies were counted. Incubations of *E. coli* with chitosan and without CTSGs were used as positive and negative controls, respectively. Compared with the case of the negative control, inhibitions of the growth of *E. coli* in the cases of chitosan and all CTSGs were statistically significant (*p* < 0.001). Several mechanisms for the inhibition of microbial growth by chitosan have been reported. The most appropriate mechanism is the interaction between the positive charge of chitosan and negatively charged membranes of microbial cells [38,39,40]. The amino groups on the chitosan backbone are protonated and positively charged. The positively charged amino groups (NH_3_^+^) interact with the negatively charged cell membranes of the microorganism via electrostatic forces, resulting in antimicrobial effects. This interaction alters the permeability of the cell wall of the microorganism, disrupts the intracellular osmotic balance, and ultimately inhibits microbial growth. Moreover, this interaction causes the hydrolysis of the peptidoglycans in the microbial wall. Consequently, intracellular electrolytes (for instance, Na and K ions) and various low-molecular-weight components (for example, lactate dehydrogenase, glucose, nucleic acids, and proteins) leak out. This prevents the biosynthesis of the microbial cell wall and blocks the transport of substances in and out of the cell wall, leading to antimicrobial activity. In the cases of CTSGs, the unreacted amino groups interacted with the membranes of microbial cells, resulting in antimicrobial effects.

The number of colonies in the case of chitosan was 0 CFU/mL, suggesting the complete inhibition of the growth of *E. coli*. In the cases of CTSGs, a slight growth of *E. coli* was observed, which increased with an increase in CR. The decrease in the number of unreacted amino groups in CTSGs with an increase in CR weakened the interaction of CTSGs with the cell membranes of *E. coli*, resulting in the low antimicrobial activities of CTSGs. The antimicrobial activities of CTSG 1 and 2 were not significantly different from that of chitosan, indicating that the antimicrobial properties of CTSGs with CRs of less than 0.14 were comparable to that of chitosan.

From the above results, CTSGs exhibited antimicrobial activity against the gram-negative bacterium *E. coli*. In general, chitosan is known to have antimicrobial activity against gram-positive as well as gram-negative bacteria [41,42]. A notable difference between gram-negative and gram-positive bacteria is the cell wall structure, which consists of a thick peptidoglycan layer for gram-positive bacteria and lipopolysaccharides for gram-negative bacteria. The thick cell wall prevents chitosan from binding directly to the bacterial cell membrane. There are also differences on the cell surface; gram-negative bacteria present a more negative charge than gram-positive bacteria because lipopolysaccharides are often attached to phosphate groups. The more negatively charged cell surfaces are more likely to interact electrostatically with chitosan. That is, the microbial cell wall structure and cell surface charge have a significant influence on the antimicrobial activity. Further evaluating the antimicrobial activities of CTSGs against various microbial species is necessary in order to elucidate the mechanism for its antimicrobial activity.

## 3. Conclusions

Herein, CTSGs were successfully synthesized by crosslinking chitosan with OS under an aqueous condition without catalyst. The imine bonds formed by the reaction of the amino groups in chitosan with OS and unreacted aldehydes were converted to secondary amines and alcohols, respectively, by immersing CTSGs in aqueous NaBH_4_. CRs of CTSGs increased with an increase in the feed amount of OS during CTSG preparation. The thermal stabilities, dynamic moduli, and water absorbencies of CTSGs were considerably dependent on CR. CTSGs effectively inhibited the growth of *E. coli*, and their antimicrobial activities were dependent on CR. As the CTSGs prepared in this study are composed of carbohydrates only, they can be applied in the food, medical, and pharmaceutical fields. For example, CTSGs can potentially be used as antimicrobial wound-dressing gels; nevertheless, further research, for instance, on the ecological safeties, the biological safeties, the cell toxicities, and antimicrobial activities of CTSGs against other microorganisms, are required in this regard. Additionally, the unreacted amino and hydroxyl groups of chitosan can be easily modified, endowing CTSGs with various functionalities, such as drug loading and release capacities for application as DDS carriers [43], and antioxidant properties for application in food preservation [44].

## 4. Materials and Methods

### 4.1. Materials

Chitosan (average molecular weight = 1 × 10^5^ according to manufacturer’s data), with a deacetylation degree of 100%, was obtained from Dainichiseika Color & Chemicals Mfg. Co., Ltd. (Tokyo, Japan). Sucrose, sodium periodate, and barium chloride dihydrate were purchased from Fujifilm Wako Pure Chemical Co., Ltd. (Osaka, Japan). NaBH_4_ was procured from Tokyo Chemical Industry Co., Ltd. (Tokyo, Japan). Acetate buffer was prepared using analytical-grade sodium acetate and acetic acid purchased from Kanto Chemical Co., Inc. (Tokyo, Japan).

### 4.2. Preparation of OS

OS was fabricated using a previously described method [23]. Briefly, sucrose (6.0 g, 17.5 mmol) and sodium periodate (11.3 g, 52.5 mmol) were dissolved in deionized water (200 mL), and the resulting mixture was stirred at 298 K for 26 h. After the reaction, barium dichloride (6.42 g, 26.2 mmol) was added to the resulting mixture followed by stirring at 278 K for 1 h to allow the complete precipitation of barium iodate. The resulting solution was filtered, and the supernatant solution was passed through a cation-exchange column filled with an anion-exchange resin (Amberlite FPC3500, Organo Co., Ltd., Tokyo, Japan) adjusted to the OH type followed by passing through a cation-exchange column filled with a cation-exchange resin (Amberlite IRA402BLCl, Organo Co., Ltd., Tokyo, Japan). OS was obtained as a white solid powder from the lyophilized solution.

Concentrations of OS(I) and OS(II) in OS were 52 and 48 mol%, respectively. The molecular formula of the OS mixture (C_12_H_18_O_11_)_0.52_ (C_11_H_16_O_10_)_0.42_ was C_11.58_ H_17.16_ O_15.58_. The aldehyde content per unit mass of OS was 12.3 mmol g^−^^1^.

### 4.3. Synthesis of CTSGs

Chitosan (0.3 g, 1.9 mmol of an average monomer unit) was completely dissolved in 2 wt% aqueous acetic acid (7.5 mL), followed by the addition of OS (30 mg) dissolved in deionized water (2.5 mL) under stirring at 298 K for 10 min. The reaction solution was allowed to stand at 298 K for 24 h to facilitate crosslinking. Then, the reaction mixture was crushed and soaked in 2 wt% aqueous NaBH_4_ to reduce the formed imine bonds and unreacted aldehyde moieties. After leaving the reaction mixture in aqueous NaBH_4_ for 24 h at 298 K, the resulting mixture was subjected to dialysis using a dialysis membrane tube with a molecular weight cutoff of 12,000 (Thermo Fisher Scientific, Waltham, MA, USA) against deionized water followed by lyophilization to achieve CTSG 1. CTSGs with different chitosan and OS concentrations were prepared using a similar method (Table 1).

### 4.4. Structural Characterization

Attenuated total reflectance (ATR)-FTIR spectra were obtained using an ALPHA II FTIR spectrometer (Bruker Optics GmbH & Co., KG, Karlsruhe, Germany) at 295 K. To improve the signal-to-noise ratio for each spectrum, 32 interferograms with a spectral resolution of ±4 cm^−1^ were averaged. Background spectra, which were acquired under identical conditions, were subtracted from the sample spectra.

Solid-state ^13^C NMR (SSNMR) spectroscopy was performed at 298 K using an AVIII500 spectrometer (^1^H frequency of 500.13 MHz) equipped with a dual-tuned 4 mm magic-angle spinning probe (Bruker BioSpin GmbH, Karlsruhe, Germany). The sample (80 mg) was packed into a 4 mm ZrO_2_ rotor, and the rotation frequency of the rotor was set to 10 kHz. The SSNMR spectra were obtained using ^13^C-excitation pulse length (flip angle of 30°), and the data acquisition and repetition times were fixed at 1.5 μs, 20 ms, and 30 s, respectively. The ^13^C chemical shifts were calibrated using the carboxyl carbon resonance of D-glycine (176.03 ppm) as an external standard.

### 4.5. TG/DTA

TG/DTA curves were acquired via an EVO2 TG 8120 Plus thermogravimetric dynamic thermal analyzer (Rigaku Co., Tokyo, Japan) using an Al_2_O_3_ crucible under a nitrogen gas flow. Each sample weighed 15 mg, and the thermograms were obtained in the 50–500 °C temperature range at a heating rate of 5 °C min^−1^.

### 4.6. Water Absorbency

Water absorbencies were measured via gravimetric analysis. Each sample of a fixed weight was separately immersed in a large amount of acetate buffer with different pH values of 3, 4, and 5 at 298 K for 24 h. Thereafter, the swollen sample was centrifuged at 8000× *g* for 10 min. The equilibrium water absorbency of the sample was determined using Equation (3):

(3) $$Water\;absorbency=\frac{m_{2}-m_{1}}{m_{1}},$$

where *m*_1_ and m_2_ are the weights of the samples in the dry and swollen states, respectively. All experiments were conducted in triplicate, and the results were averaged.

### 4.7. Rheological Measurements

The rheological behavior of each sample swollen and saturated with 20 mM acetate buffer (pH = 4) at 298 K for 24 h was evaluated in triplicate using a Physica MCR 301 rheometer (Anton Paar GmbH, Graz, Austria) equipped with a 25 mm parallel-plate measuring geometry, Peltier device for temperature control, and Rheoplus 32 data analyzer. The gap and strain were set to 1.0 mm and 0.5%, respectively. The oscillatory shear responses at 298 K were determined under 0.1 Pa over the frequency range of steady shear tests with an angular frequency range of 10^−1^–10^2^ rad∙s^−1^.

### 4.8. Determination of Antimicrobial Activities

*E. coli* NBRC3301 cultured on standard method agar plates (Nissui Pharmaceutical Co., Ltd., Tokyo, Japan; Composition: 0.25 *w*/*v*% yeast extract, 0.5 *w*/*v*% peptone, 0.1 *w*/*v*% glucose, and 1.5 *w*/*v*% agar) at 310 K was used to evaluate the antimicrobial activities of CTSGs. The single colony was cultured in LB medium (MP Biomedicals, Santa Ana, CA, USA; Composition: 1.0 *w*/*v*% tryptone, 0.5 *w*/*v*% yeast extract, and 1.0 *w*/*v*% NaCl) for 24 h at 310 K, and the resulting colonies were suspended in saline solution (Otsuka Pharmaceutical Factory, Inc., Tokushima, Japan) to achieve an optical density at 600 nm (OD_600_) of ca. 0.2. The suspension diluted 10^3^-fold in fresh LB medium (1 mL) was separately added to a 24-well microplate containing CTSGs (20 mg dry weight), neat chitosan as a positive control, and no sample as a negative control. The bacterial culture was used at a concentration of 0.38 × 10^4^ CFU/mL, which was determined by counting the number of colonies after the incubation of the bacterial suspension on a standard method agar plate at 310 K for 24 h. After incubation at 310 K for 3 h, the supernatant of the culture medium (0.1 mL) was diluted 10-fold with saline solution and then cultured on the standard method agar plate at 310 K for 24 h. The antimicrobial activities of the samples were determined by counting the number of colonies on the plate. The values are presented as the mean of the results of three identical samples (n = 4). Statistical analyses were performed using Tukey’s test using analysis of variance with R (EZR version 1.61, Jichi Medical University, Saitama, Japan) [45].

## Figures and Tables

**Table 1:** Initial feed amounts of chitosan and oxidized sucrose (OS) for the preparation of chitosan hydrogels (CTSG 1–3). Degrees of crosslinking (CRs) of CTSGs are also presented.

Sample	Initial Feed Amount		Feed Molar Ratio of Aldehyde/Amine	CR ^a^	*n_OS_* ^b^
	Chitosan (g) (Amine (mmol))	OS (g) (Aldehyde (mmol))			
CTSG 1	0.30 (1.9)	0.30 (0.4)	0.2	0.09	0.04
CTSG 2	0.30 (1.9)	0.45 (0.6)	0.3	0.14	0.06
CTSG 3	0.30 (1.9)	0.75 (0.9)	0.5	0.20	0.11

^a^ Number of amine bonds formed between chitosan and OS per glucosamine residue. ^b^ Number of OS molecules reacted per glucosamine residue.

## Data Availability

The data presented in this study, supporting the results, are available in the main text. Additional data are available upon request from the corresponding authors.

## Supplementary Materials

The following supporting information can be downloaded at: https://www.mdpi.com/article/10.3390/gels9100786/s1, Figure S1: (a) Initial (*T_i_*) and (b) final (*T_f_*) degradation temperatures for the second degradation of CTSG 1–3 and chitosan determined by thermogravimetric analysis.
