# Supplementary material for: Chitosan Hydrogels Crosslinked with Oxidized Sucrose for Antimicrobial Applications
Source: Gels. 2023 Sep 29;9(10):786. doi: 10.3390/gels9100786 (PMC10606239; doi:10.3390/gels9100786)
Supplement: Supplementary file 1 [file gels-09-00786-s001.zip › gels-2610425-supplementary.pdf]

gels

MDPI

Supplementary material

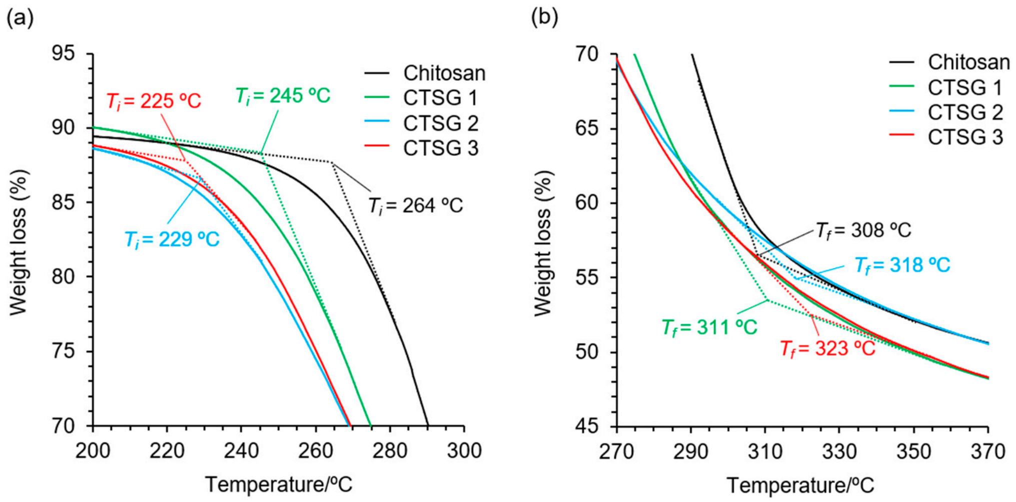

Figure S1. (a) Initial (T_i) and (b) final (T_f) degradation temperatures for the second degradation of chitosan hydrogels (CTSG 1–3) and chitosan determined by thermogravimetric analysis.
